# Infection and Inflammation in Schizophrenia and Bipolar Disorder: A Genome Wide Study for Interactions with Genetic Variation

**DOI:** 10.1371/journal.pone.0116696

**Published:** 2015-03-17

**Authors:** Dimitrios Avramopoulos, Brad D. Pearce, John McGrath, Paula Wolyniec, Ruihua Wang, Nicole Eckart, Alexandros Hatzimanolis, Fernando S. Goes, Gerald Nestadt, Jennifer Mulle, Karen Coneely, Myfanwy Hopkins, Ingo Ruczinski, Robert Yolken, Ann E. Pulver

**Affiliations:** 1 McKusick Nathans Institute of Genetic Medicine, Johns Hopkins University School of Medicine, Baltimore, MD, United States of America; 2 Department of Psychiatry, Johns Hopkins University School of Medicine, Baltimore, MD, United States of America; 3 Department of Epidemiology, Rollins School of Public Health, Emory University, Atlanta, GA, United States of America; 4 Department of Human Genetics, Emory University School of Medicine, Atlanta, GA, United States of America; 5 Bloomberg School of Public Heath, Johns Hopkins University School of Medicine, Baltimore, MD, United States of America; 6 Department of Pediatrics, Johns Hopkins University School of Medicine, Baltimore, MD, United States of America; University of Iowa Hospitals & Clinics, UNITED STATES

## Abstract

Inflammation and maternal or fetal infections have been suggested as risk factors for schizophrenia (SZ) and bipolar disorder (BP). It is likely that such environmental effects are contingent on genetic background. Here, in a genome-wide approach, we test the hypothesis that such exposures increase the risk for SZ and BP and that the increase is dependent on genetic variants. We use genome-wide genotype data, plasma IgG antibody measurements against *Toxoplasma gondii,* Herpes simplex virus type 1, Cytomegalovirus, Human Herpes Virus 6 and the food antigen gliadin as well as measurements of C-reactive protein (CRP), a peripheral marker of inflammation. The subjects are SZ cases, BP cases, parents of cases and screened controls. We look for higher levels of our immunity/infection variables and interactions between them and common genetic variation genome-wide. We find many of the antibody measurements higher in both disorders. While individual tests do not withstand correction for multiple comparisons, the number of nominally significant tests and the comparisons showing the expected direction are in significant excess (permutation p=0.019 and 0.004 respectively). We also find CRP levels highly elevated in SZ, BP and the mothers of BP cases, in agreement with existing literature, but possibly confounded by our inability to correct for smoking or body mass index. In our genome-wide interaction analysis no signal reached genome-wide significance, yet many plausible candidate genes emerged. In a hypothesis driven test, we found multiple interactions among SZ-associated SNPs in the HLA region on chromosome 6 and replicated an interaction between CMV infection and genotypes near the *CTNNA3* gene reported by a recent GWAS. Our results support that inflammatory processes and infection may modify the risk for psychosis and suggest that the genotype at SZ-associated HLA loci modifies the effect of these variables on the risk to develop SZ.

## Introduction

Schizophrenia (SZ) and bipolar disorder (BP) are debilitating chronic psychiatric diseases, each affecting approximately 1% of the world’s population. Both disorders are clinically and etiologically heterogeneous. Studies have demonstrated significant heritability estimated to be around 80% [1]. Twin concordance of both disorders is around 50% [2,3] therefore non-genetic factors also contribute significantly. The most consistently identified environmental risk factors for SZ include winter birth, significant maternal malnutrition, obstetric complications, migrant status, urban environment, cannabis use and a variety of infections [4]. In addition to epidemiological similarities between SZ and BP and the similarly high heritability, many studies including recent genome wide association studies (GWAS) suggest common genetic underpinnings [5,6].

GWAS have now begun to identify specific variants and genes that increase the risk for SZ [7] and point to shared variants with multiple disorders [8]. This success is accompanied by the realization that, as with other complex disorders [9], much of the heritability will not be explained by the additive effects of common variants. Among many explanations for this is the presence of interactions between genes or between genes and the environment [9]. The environment can have a major influence on heritability, as changes can make existing, previously neutral variants become contributors to the risk [10].

Infection and immune response have been studied in SZ across two centuries [11], and through a variety of study designs many infectious agents have been associated with SZ risk [12], including *Toxoplasma gondii* (TOXO), Herpes simplex virus type 1 (HSV1), cytomegalovirus (CMV) and human herpes virus 6 (HHV6) [13]. In more recent literature, studies have focused on first episode and drug-naïve patients reporting similar results [14]. The diverse list of infectious agents suggests that the associations might stem from the response to infection and immune activation rather than the specific infectious agents. Interestingly, the list of infection and immunity-related factors has recently expanded to include antibodies against food antigens such as gliadin [15]. Additionally, C-reactive protein (CRP), a pentameric protein of the pentraxin family used in clinical practice as a non-specific marker of tissue injury, infection and inflammation, has also been reported elevated in plasma from SZ patients [16] including findings from a recent meta-analysis [17] and patients not talking psychotropic medication [18]. In addition to SZ, infection has also been implicated in BP [19], including associations with anti-CMV and anti-TOXO antibodies and antibodies to food antigens such as gliadin (anti-GLD) [20–23], although not all associations have been consistent [24]. The involvement of infection and immune activation in SZ and BP raises the possibility that genetic variants that influence the susceptibility or immune response to certain infections may determine whether an individual exposed to the infectious agent has higher risk or not. This genotype by infection interaction hypothesis, has been supported by animal models [25] but, to our knowledge, has only been tested in one prior genome wide study in humans study for CMV infection in SZ [26]. That study identified only one promising signal for genotype by infection interaction.

The most recent published SZ GWAS has now reported on over 100 SZ-associated loci [7]. The most consistent and strongest association across studies, is that of SNPs in the human leukocyte antigen (HLA) region which is important for immune response but also for synaptic plasticity, [27] and therefore an obvious candidate for interactions with antigen exposure in determining SZ risk.

The Epidemiology-Genetics program in psychiatry (EpiGen) at Johns Hopkins University under the leadership of AEP has been collecting individuals affected with SZ and BP and their families for the last 30 years. Since 1996, recruitment has focused on Ashkenazi Jewish (AJ) individuals in the U.S affected with SZ or BP, their families and screened controls. In addition to the extensive information available for the research participants, plasma collected at the time of examination was available for a significant fraction of this sample. Genome wide genotype data was also previously obtained and available. Here we report on our analyses of the plasma of these individuals for anti-TOXO, anti-HSV1, anti-HHV6, anti-CMV and anti-GLD, as well as for CRP. For details on each measurement see Table 1 and the corresponding references [14,28,29] We compare frequencies or levels of seropositivity (depending on the variable) in patients, their parents and healthy controls and perform an analysis for their interactions with genetic variation across the genome, a search for DNA variants where the effect of infection on the risk differs by across genotypes. Our work supports a role of infection, immune response and inflammation in psychiatric disease, replicates a reported genotype-infection interaction and points to new ones that warrant further examination.

**Table 1:** Infection and inflammation related variables explored in this study.

Antibodies	Assay	Purpose of test	Proposed relevance to SCZ and BP (based on current literature)
TOXO	ELISA detecting IgG antibodies against Toxoplasma *gondii*	An indicator of past infection with this persistent parasite. IgG may also increase upon reactivation of existing infection, or infection with another serotype.	Infection modulates neurotransmitters, including dopamine; and seropositivity is associated with SCZ and BP.
HSV1	ELISA to detect IgG antibodies to Herpes Simplex virus type 1	An indicator of past and current infection. Levels increase upon reactivation of latent infection.	Infection or titers associated with cognitive deficits and neuroimaging findings in SCZ and BP.
CMV	ELISA detecting IgG antibody to Cytomegalovirus	An indicator infection. Levels increase upon reactivation of this latent infection.	Higher titers correlate with cognitive deficits of SCZ and BP, though a diagnosis of SCZ may correlate with lower titers.
HHV6	ELISA detecting IgG antibody Human Herpesvirus 6	Indicates infection with this persistent neurotropic virus.	May be negatively or positively correlated with SCZ prevalence, perhaps due to stage of infection, virus type (HHV-6A or HHV6B), or immunogenetic factors.
GLIADIN	Measures IgG antibodies to gliadin, a gluten protein	Increases in antibodies to gliadin are found in celiac disease.	Increased rate of celiac disease in schizophrenia, which may inform the autoimmune component of SCZ.
Acute phase reactant			
CRP	Sandwich-based ELISA for quantitation of human C-reactive protein levels	Higher levels indicates chronic inflammatory state. May also reflect neuroinflammation.	CRP levels are significantly increased in SCZ and unipolar depression, and possibly BP.

See text for references on the relevance to SCZ and BP.

## Methods

### 1. Subjects

Subjects included research participants with a diagnosis of SZ (including schizoaffective) or BP diagnosis recruited over a 15-year period (1996–2011) through advertisements, talks, letters to leaders service providers of the Jewish community and a study web site. The patients were interviewed in-person and diagnosed through a consensus procedure described in detail elsewhere [30,31]. Approximately 17% of the SZ patients were diagnosed with schizoaffective disorder. Screened controls were recruited over a four-year period (2003–2007) at Jewish community professional meetings, community centers and synagogues. All cases and controls were ascertained reporting four grandparents of known AJ descent. Parents were also examined in person by a doctoral-level clinical psychologist. Examiners were blind to the subject’s diagnosis; they did not know whether the family was being assessed for the study of BP or SZ/SZA. Most of the subjects were seen in their homes. Detailed clinical methods are available from prior publications [31,32] The frequency of a positive diagnosis in the parents was under 10%. It is therefore likely that some of the parents are also patients, yet the impact of this small group to overall statistical differences would be very small. Blood for DNA and plasma from cases parents and controls were collected and kept frozen at -18°C.

The Johns Hopkins institutional review board approved the recruitment methods, protocols and informed consent documents. All participating human subjects provided written informed consent to participate in this study. If the subject was a minor, written informed consent was obtained from both the subject and his/her parent or legal guardian (if subject was age 15–18) or written informed consent was obtained from the parent or legal guardian and an assent procedure was completed with the subject. (if the subject was under age 15). If the subject was not capable of consent, written informed consent was obtained by a legal guardian.

For the current study we had plasma samples and successfully obtained antibody (Ab) and CRP data (which we refer to collectively as serology data) for 580 AJ SZ probands, 262 fathers of SZ probands, 266 mothers of SZ probands, 489 AJ BP probands, 314 fathers of BP probands, 314 mothers of BP probands, and 362 AJ controls.

### 2. Immunoassay Measurements

We measured plasma IgG class Abs anti-HSV1, anti-HHV6, anti-CMV, anti-TOXO, and anti-GLD using previously described immunoassay methods [33]. Briefly, diluted plasma was applied to antigens immobilized on the wells of microtiter plates and bound Ab was quantified by means of reaction with enzyme-labeled anti-human IgG and the corresponding substrate. Reagents and assay kits for anti-HSV1 were obtained from Focus Laboratories, Cypress, CA. Reagents for anti-HHV6 were obtained from Advanced Biotechnologies Incorporated, Columbia, MD. Reagents for anti-CMV, anti-TOXO, and for the measurement of CRP were obtained from IBL Laboratories, Hamburg, Germany. Reagents for anti-GLD were obtained from Inova Diagnostics, San Diego, California.

SZ and BP cases, parents and controls were randomly distributed on 32 plates. Ab in the plasma specimen was quantified by the measurement of colorimetric substrate by means of a microplate colorimeter and converted into a ratio by dividing the amount of color generated in the sample wells by the amount of color generated from reaction with a weakly positive sample provided by the manufacturer. For comparison of quantitative results among the different Ab assays, this ratio was standardized to a value of 1.0 on each microtiter plate.

### 3. Immunoassay data cleaning

**Fig 1:**
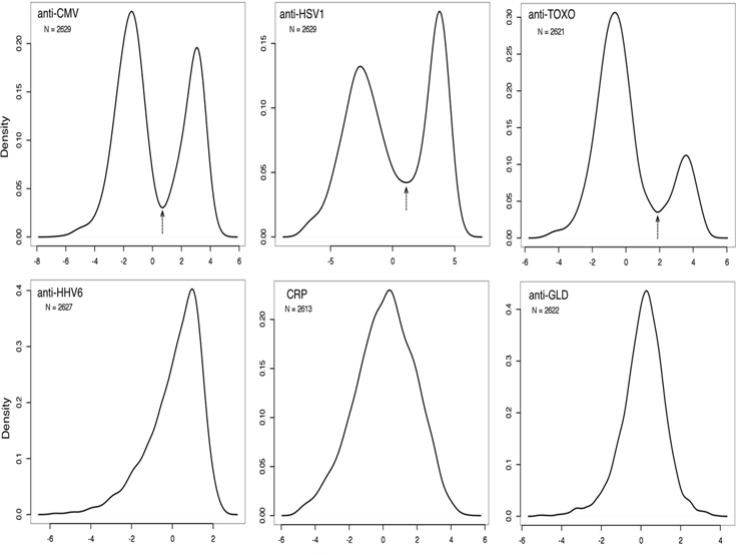
Density plots showing the distribution of the log-transformed residuals of our variables. Anti-CMV, anti-HSV1 and anti-TOXO show clearly bimodal distributions and were transformed to binary variables. An arrow indicates the transformation threshold.

Data for analyses included 2660 records of subjects’ plasma sample assay results. One of the 32 plates showed significant measurement distribution differences from the rest and was excluded as a technical failure. The R Statistical software package “lme4” was used to run linear mixed effect models on the 6 assay variables controlling for the effects of assay processing plate and plasma storage years (with plate as the random effect and storage years as the fixed effect). Assay variable values of zero were removed as failed assays, the model was applied to the log_2_ transform of each of the 6 assay variables, and residuals from each were saved for analysis. The density plots for all residuals are shown in Fig. 1. Three of the measurements—anti-GLD, anti-HHV6 and CRP—showed continuous near-normal distributions, probably as a reflection of the high rate of corresponding exposures, so no further transformation was performed. The remaining, anti-HSV1, anti-CMV and anti-TOXO showed clearly bimodal distributions (Fig. 1). For these, a threshold was chosen based on the lowest point between the two density plot peaks and they were converted to binary variables.

### 4. Genotyping

DNA from blood was extracted with the Gentra Puregene Kit or the QIAGEN DNeasy Blood and Tissue Kit. Genotyping was performed with the Affymetrix Human Genome-Wide SNP Array 6.0 at Emory University and has been previously described [34]. Genotypes were called using the corrected robust linear mixture model (CRLMM) [35], an algorithm for preprocessing and genotype calling of Affymetrix SNP array data. This algorithm, available as an open source Bioconductor (www.bioconductor.org) package, implements a multilevel model for genotype calls that adjusts for batch effects, avoiding biases and errors from processing different batches of samples at different times.

### 5. Genotype data cleaning

Genotype data cleaning was performed using the software package PLINK [36]. We followed the cleaning steps suggested by the Psychiatric GWAS consortium (PGC) [37]: 1) remove SNPs with more than 5% missing data, 2) remove subjects with more than 2% missing data after the first step of SNP removal 3) remove SNPs with more than 2% missing data after the removal of the subjects 4) remove SNPs out of Hardy Weinberg equilibrium at p < 10^-6^ to account for the multiple tests. We further excluded SNPs with minor allele frequency < 0.05 as underpowered in our relatively small sample, likely to introduce artifacts in the presence of even small deviations of the traits from normality. The final dataset included data on 516,638 SNPs for 460 SZ cases, 397 BP cases and 241 controls with serology data.

### 6. Statistical analyses

Statistical analyses were performed using R. The serology outcomes were first tested for correlations with sex and age at the time of blood draw. As these variables, and in particular the age at the time of blood draw, showed significant correlations with multiple measurements they were included as covariates in all further analyses. Comparisons between case control and parent groups were made using generalized linear models with serology as the outcome and predictors that included group status, age at blood draw and sex in linear or logistic models depending on the outcome variable.

GWAS analyses were performed using PLINK. Gene x infection interaction tests were performed with the respective disease phenotype (SZ or BP) as outcome and genotype, sex, age at blood draw, serology result and the genotype x serology interaction factor as predictors. The results for the interaction factor were then explored for significant signals. The results for associations of genotypes with the disease phenotype will be reported separately.

Permutation tests to determine the significance of the excess of signals and their direction were performed using scripts in perl and followed by analysis in R under identical parameters as the primary analysis. The scripts randomized the link between "status" (BP, SZ, control, father, mother) and Ab variables keeping the parameter "age at blood draw" linked to the Ab variables, where it is relevant. Specifically the data table was separated vertically in two, one retaining the Ab variables together with age at blood draw and the other the "status". One of the two sub-tables was randomly re-ordered and they were merged again for analysis. Additionally, because sex is relevant to both the Ab variables and status before the randomization described above we separated the dataset horizontally into male and female subsets and merged it again before analysis, thus performing a within-sex randomization where a females' Ab measurements always come from other females and all status categories retain their sex distribution. We performed 3,000 permutations to test the significance of signal enrichment in Table 2 and 5,000 permutations to test the significance of the multiple significant interactions in the HLA region (see below). Our scripts are available upon request.

**Table 2:** Effect size (beta) and standard errors of effects of 5 antibodies and CRP.

Antibodies	SZ patients	SZ mothers	SZ fathers	BP patients	BP mothers	BP fathers
	β ± S.E (p)	β ± S.E (p)	β ± S.E (p)	β ± S.E (p)	β ± S.E (p)	β ± S.E (p)
TOXO	-0.02±0.2 (n/a)	0.30±0.23 (0.095)	0.09±0.25 (n.s.)	0.04±0.21 (n.s.)	0.1±0.23 (n.s.)	0.04±0.25 (n.s.)
HSV1	-0.20±0.16 (n/a)	0.23±0.19 (n.s.)	0.43±0.22 **(0.027)**	0.24±0.16 (0.07)	0.48±0.18 **(0.005)**	0.41±0.21 **(0.026)**
CMV	-0.01±0.17 (n/a)	0.19±0.2 (n.s.)	0.65±0.23 **(0.002)**	0.26±0.17 (0.06)	0.19±0.19 (0.06)	0.48±0.22 **(0.015)**
HHV6	0.12±0.09 (0.07)	0.17±0.11 (0.07)	0.20±0.14 (0.07)	0.19±0.09 **(0.026)**	0.06±0.11 (n.s.)	0.12±0.14 (n.s.)
GLIADIN	0.07±0.08 (n.s.)	-0.10±0.10 (n/a)	0.02±0.12 (n.s.)	0.04±0.08 (n.s.)	0.07±0.10 (n.s.)	0.04±0.11 (n.s.)
Markers	SZ patients	SZ mothers	SZ fathers	BP patients	BP mothers	BP fathers
CRP	1.04±0.12 **(1.2E-16)**	0.25±0.16 (0.06)	0.05±0.17 (n.s.)	0.42±0.14 **(0.0013)**	0.44±0.15 **(0.002)**	0.20±0.16 (n.s.)

All comparisons are with controls. P-values are 1-tailed; only p<0.1 are shown; nominally significant p-values are in bold; n.s. = not significant; n.a. not applicable because direction is opposite from the expected.

The complexity of our analyses requires some clarifications with regard to multiple comparisons. In the GWAS section we performed six independent GWAS one for each measured variable and a p-value of below 8.3x10^-9^ would be necessary to declare significance. In comparing the case and parent groups with controls (Table 2) we performed 36 comparisons (6 variables x 6 groups) and a p-value <0.0013 would be necessary. In the latter when we refer to nominal significance we mean a p-value < 0.05 and to suggestive results a p-value < 0.1. The remaining tests including testing for signal enrichment across all Ab comparisons, testing for excess interactions at the HLA locus and replicating reported interactions and associations are single hypothesis based tests and we consider a p-value of 0.05 significant.

## Results

We first investigated the effects of the age of the individual at the time the blood sample was drawn and of the subject's sex on the Ab and CRP measurements. For this analysis we used the entire sample correcting for diagnostic category or parent status. We found a strong positive correlation with age at the time of blood draw for all the measurements with the exception of anti-HHV6 where a strong negative correlation was observed (all tests with p < 10^-3^). We also found a trend for lower levels in males, however this was only statistically significant for anti-HHV6 and anti-CMV (p = 2.45x10^-10^ and p = 0.016 respectively). Sex and age at blood draw were subsequently used as covariates in all analyses.

### 1. Antibody levels in patients, parents and controls

Prompted by the literature and the available materials and data we tested whether the patients and their parents have higher seropositivity rates (or Ab levels) than controls for any of our serology measurements. Our results are summarized in Table 2. All tests are 1-sided as only increases are in line with our hypothesis. Among Ab measurements we observed 6 nominally significant differences (in bold) but none could withstand Bonferonni correction. Of the 30 comparisons, 26 where in the expected direction. In order to determine whether this number of positive results and directional consistency represents a deviation from the null hypothesis, we performed permutations as described in the methods. The probability of observing 6 or more nominally significant 1-sided p-values in 3,000 permutations was 0.019. The probability of observing 26 or more of the 30 comparisons in the expected direction 3,000 permutations was 0.004.

### 2. CRP levels

In contrast to the individually modest results for infectious agents and gliadin, the comparisons for CRP showed highly significant differences (Table 2). CRP levels, adjusted for age and sex, were higher in both SZ and BP. They were also significantly higher in the mothers of the BP patients and showed a trend in the mothers of the SZ patients, but not in the fathers.

### 3. GWAS for genotype by Ab interactions

We performed a genome wide association analysis for interactions between each plasma variable and genotype by including an interaction term in the logistic regression model. The aim of this GWAS was not to identify loci predisposing to SZ or BP, for which we knew the sample size was underpowered, but to identify genetic variants that modify the relationship between infection or immune reaction to an antigen and the risk for psychosis. While the effect sizes of common risk variants is expected to be small, due in part to the negative selection of stronger effect alleles, this might not be the case for interactions. The results on the interaction terms are shown in Table 3. The primary results of the GWAS for SZ will be reported elsewhere.

**Table 3:** Genes showing suggestive interactions with antibody levels.

Phenotype	Interactor	SNP ID	Chr.	position (hg19)	p-value	Refseq genes within 200 Kb
BP	anti-CMV	rs6479352	9	94281709	8.1E-06	ROR2, **NFIL3**, AUH, MIR3910
BP	anti-CMV	rs9910816	17	69281765	2.0E-06	none
BP	anti-HSV1	rs885553	2	45456389	7.9E-07	LINC01121, SRBD1
BP	anti-HSV1	rs4234848	4	80850804	3.6E-06	ANTXR2, PCAT4
BP	anti-HSV1	rs3736986	9	36194568	6.2E-06	**CLTA**,GNE,RNF38,GLIPR2,RECK
SZ	anti-CMV	rs11871847	17	66344702	6.1E-06	CASC17
SZ	anti-HSV1	rs10170846	2	223517704	2.3E-06	FARSB, MOGAT1, SGPP2
SZ	anti-HSV1	rs12369635	12	129561355	8.3E-06	TMEM132D, GLT1D1
SZ	anti-TOXO	rs1009840	6	134546684	8.1E-06	**SGK1**, SLC2A12

Genes with significant functional evidence are in bold (see text).

None of the 6 tested variables in either disease phenotype provided a genome wide significant result after accounting for multiple testing. However, we identified suggestive associations at p < 10^-5^ for anti-CMV and anti-HSV1 in both disorders and for anti-TOXO in SZ, shown in Table 3 along with the nearby genes. We only report these as candidates, whose validity needs to be examined and independently replicated. Overall we see no signal inflation as with the ~500,000 tests performed, we expect ~5 SNPs for each phenotype to yield a p < 10^-5^ under the null hypothesis.

### 4. Replication of a GWAS reported interaction

We examined in our GWAS results the SNP rs7902091 recently reported to show interactions with CMV in the only other published genotype by infection interaction GWAS for SZ by Borglum et al [26]. In our data, rs7902091 replicated the reported interaction with a p-value of 0.047 in the same direction as reported.

### 5. Interactions with SZ-associated HLA variants

Due to the importance of HLA in immune responses and the strong associations of DNA variation in the HLA region with SZ, we explored these SZ-associated variants in our data for evidence of interactions between genotype and Ab titer or positivity. We selected the SZ-associated markers (p < 10^-8^) from the largest meta-analysis recently reported by the PGC [7] and identified 116 HLA region SNPs that were also genotyped in our dataset. When we explored the results of these SNPs in our interaction analysis we found them unremarkable, except for anti-HSV1 where there were 46 SNPs (38%) that showed nominally significant interactions. We considered that the extraordinarily high LD in the HLA region might account for this result; however, when we explored the LD between these SZ-associated variants we found that although this was sometimes the case, often their genotype correlations were modest or zero (S1 Fig). Encouraged by this and in order to calculate whether our results deviate significantly from the expectation given the LD, we proceeded to test how often we would observe 46 or more nominally significant interactions among those SNPs when permuting the phenotype as described in the methods. In 5,000 permutations we only observed that 5 times (p = 0.001). This result remains significant after correcting for testing 6 inflammation variables (corrected p = 0.006) and suggests that many of the observed interactions are fully or partially independent and that there is a strong enrichment of such interactions among HLA region SZ-associated SNPs. The SNPs showing significant interactions are shown in S1 Table. In every case the combination of the SZ risk allele and infection resulted in higher risk. The LD structure of the 116 SNP (with the 44 SNPs showing interactions highlighted) is shown in S1 Fig.

### 6. Association between a *CRP* gene SNP (rs2794520), CRP levels and SZ.

A recent GWAS has identified a strong signal close to the *CRP* gene whose genotype influences CRP levels [38]. We tested this SNP (rs2794520) in our dataset by combining cases and controls and running a quantitative trait analysis controlling for affection status. We observed robust replication of the reported association with CRP (p = 2.6x10^-4^). The same SNP showed no association with SZ in the PGC or in our dataset, or interaction with CRP in our dataset.

It must be noted that another interesting candidate that we did not examine is the IgG1 heavy chain locus on chromosome 14 whose allotypes have been associated with both HSV1 and gliadin [39,40]. Unfortunately the available data around the gene were sparse and insufficient for high quality imputations.

## Discussion

We tested the hypothesis that there are increased rates of infection and inflammation in patients with SZ or BP and their parents, which might suggest a role in disease. While individual tests were underpowered for reliable individual comparisons, the excess of nominally significant results and the almost consistent direction toward higher levels of seropositivity or Ab titers in patients and their parents support for this hypothesis. The increased seropositivity in the parents might either reflect a shared family environment or a genetically driven increase in susceptibility to infection, or Ab production, persistence or affinity.

Possible reasons for the reduced power to support individual infectious agents possibly include small sample size and perhaps noise introduced by the interval between the blood draw and the presumed relevant time point. Infection is most likely to be important during gestation, perinatally, or close to the disease onset [14,41], rather than at the time of patient recruitment, when we collected blood which represents a highly variable interval from the disease onset during which Ab levels could change significantly. The fact that we observed highly significant excess of positive results and directional consistency despite this limitation, suggests a significant association between exposure or immune reaction to infection and the risk to develop SZ or BP, which in order to be understood in its detail will require larger studies designed to address each specific question.

The strongest results came from the comparisons of CRP levels and agree with previous studies, including studies of drug free patients [18,42,43]. Note, however, that CRP is known to be associated with smoking and body mass index, both of which are known to be increased in SZ and BP patients [17], therefore these results should be considered with caution. Interestingly, we observe the same in the unaffected mothers (but not fathers) of patients, for which it is not known whether BMI and smoking are also confounders. While CRP is most often studied for its acute fluctuations, it is possible that our observation reflects a long lasting increase after a maternal exposure during pregnancy or perinatally. Another possibility is that the mother has a genetic predisposition to exaggerated pro-inflammatory responses affecting the fetus during gestation and observable throughout life. The same predisposition might also be inherited to the offspring leading to both higher CRP and increased risk for psychosis. Our results suggest that CRP studies that account for BMI and smoking should extend to SZ and BP parents as there might be important clues to the etiology of the diseases.

In this study we confirmed the reported association of SNP rs2794520 [38] with CRP levels, however we did not observe increased SZ prevalence in high-CRP allele C carriers in our or the highly powered PGC data (in fact the PGC data showed an opposite trend at p = 0.046). This suggests that if the relationship of CRP and SZ is true as others show [17], it is likely not causal but a reflection of a common underlying factor such as inflammation.

Our genome wide analysis for interactions between infection and genetic variation provided modest results, not reaching genome wide statistical significance. The top hits, however, likely contain true signals, particularly those within or near genes of strong biologically plausibility. With regard to interactions increasing the risk for SZ, the most interesting is *SGK1* on chromosome 6, far from the HLA region, which emerged for an interaction with anti-TOXO. It encodes an mTORC2-dependent regulator of the differentiation and function of T cells [44], which additionally contributes to the regulation of diverse cerebral functions [45] and acts as a mediator of cortisol effects on neurogenesis [46]. With regard to BP, the interaction between *NFIL3* on chromosome 9 and anti-CMV titers is intriguing. This gene encodes for an interleukin 3 regulated transcription factor known as E4BP4 thought to act downstream of IL-15 signaling [47]. Nfil3-deficient mice lack natural killer cells [48]. Its expression is reduced by lithium [49] and it has an established role in circadian oscillation [50], making it an excellent candidate for involvement in BP. Another interesting gene in a different region of chromosome 9 showing interaction with anti-HSV1 is *CLTA* coding for Clathrin Light Chain. Clathrin is a major player in endocytosis, important both for neuronal and immune cells [51] and likely has a role in psychosis [15].

While not all these signals are true positives and some of the functional evidence is likely coincidental, it is intriguing that many identified regions contain genes important for both the nervous and the immune system. Our goal in reporting these interactions is not to claim the discovery of variants interacting with inflammation or infection to cause disease, but to trigger replication attempts in additional samples. In the same line, we provide the first replication of a genotype by infection interaction locus in SZ, the interaction with CMV infection at the *CTNNA3* locus first reported by Borglum et al [26]. It should be noted here that as Dudbridge and Fletcher have shown [52] gene-environment dependence can lead to statistical interactions between a marker and the environment even if there is no actual interaction of the causal variant, so caution is warranted in interpreting results.

The link between infection and SZ is further strengthened by the multiple interactions we observed between HSV1 seropositivity and SZ risk alleles in the HLA region. We find multiple significant interactions with SZ-associated SNPs and our permutations suggest that they are significantly more than the null expectation and not solely due to LD, a result that easily withstands correction for the number of tests. These SZ-associated SNPs span 3.8 Mb and include multiple histone genes. Although SZ-associated SNPs were tested across the HLA region (25.4–33.5 Mb on chromosome 6), the interaction signals stop 50 Kb short of the first HLA gene, HLA-F at 29.7 Mb. As only 9 of the 116 SZ-associated SNPs overlapping between studies were located after 29.7 Mb, it is unclear whether this result should be interpreted as pointing to the specific sub-region or a consequence incomplete testing beyond that location.

In this manuscript we support the possible role of infection/inflammation in BP and SZ and we report on a GWAS for interactions with genetic variation. Further, we replicate a reported GWAS signal for CRP levels and another for an interaction between CMV infection and SNP rs7902091. Finally, we find that multiple SZ-associated SNPs in the HLA region show interactions with HSV1. These results are important and encouraging. The low statistical power of individual tests does not allow pointing to specific antibodies and the estimated effect sizes should be interpreted with caution. The results instead make evident the need to study the role of infection and inflammation in SZ and BP. Further explorations to that end could include modeling the genetic overlap between SZ and BP as it relates to infection and multivariate analyses. Further collecting materials at birth or at onset when possible, and developing large datasets through collaborative consortia would be instrumental to appropriately address these questions.

## Supporting Information

S1 Dataset File containing all the anonymized data used for the analyses in this paper with the exception of the GWAS data (due to IRB restrictions) (TXT)

S1 Dataset Info Information file explaining the data in S1 Dataset. (TXT)

S1 Fig LD structure of the 116 SZ-associated HLA region SNPs available in our data. The software Haploview was used to generate this image and the color and numbers in the diamonds are the r-squared between pairs of SNPs. The 46 SNPs that showed interactions at p<0.05 are marked with a red dot (more details are shown in S1 Table). (Note: this figure is best seen on a computer screen, as magnification is necessary to make the details legible)
(TIF)

S1 Table Details on the 46 HLA region SNPs associated with SZ and showing an interaction with HSV1 Ab. bp location corresponds to hg19; N: number informative individuals; OR: odds ratio. Many but not all of these SNPs are in strong LD (see S1 Fig).
(XLSX)
