# Supplementary figures and images for: Infection and Inflammation in Schizophrenia and Bipolar Disorder: A Genome Wide Study for Interactions with Genetic Variation
Source: PLoS One. 2015 Mar 17;10(3):e0116696. doi: 10.1371/journal.pone.0116696 (PMC4363491; doi:10.1371/journal.pone.0116696)

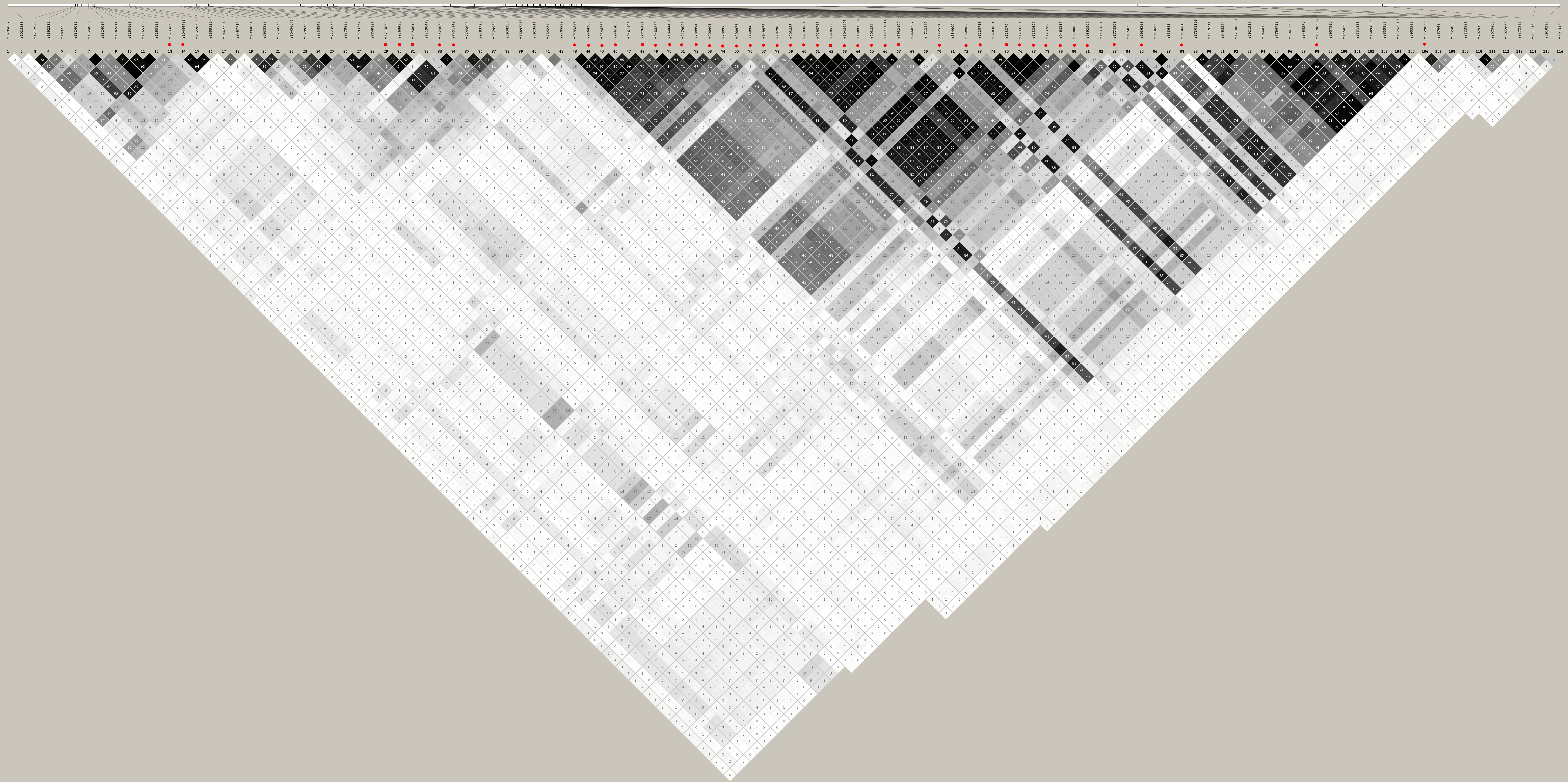

Supplement: S1 Fig — The software Haploview was used to generate this image and the color and numbers in the diamonds are the r-squared between pairs of SNPs. The 46 SNPs that showed interactions at p<0.05 are marked with a red dot (more details are shown in S1 Table). (Note: this figure is best seen on a computer screen, as magnification is necessary to make the details legible) (TIF) [file pone.0116696.s003.tif]
